# Supplementary material for: Molecular determinants of metazoan tricRNA biogenesis
Source: Nucleic Acids Res. 2019 Apr 29;47(12):6452–65. doi: 10.1093/nar/gkz311 (PMC6614914; doi:10.1093/nar/gkz311)
Supplement: gkz311_Supplemental_File [file gkz311_supplemental_file.pdf]

A

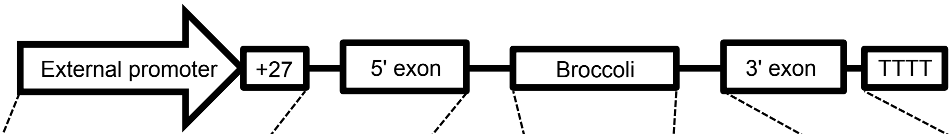

**Human reporter**	pAV U6+27 (Addgene #25709)	TRYGTA3-1	GCGGCCGC	Broccoli	GTGGCCGCGG	AGGT	TRYGTA3-1
**Heterologous reporter**	pAV U6+27 (Addgene #25709)	CR31905	AGTGCGGCCGC	Broccoli	GTGGCCGCGG	TGTTGAA	CR31905
**tricRNA reporter**	snRNA:U6:96Ab	CR31905	AGTGCGGCCGC	Broccoli	GTGGCCGCGG	TGTTGAA	CR31905
**Dual reporter**	snRNA:U6:96Ab	CR31905	AGTGCGGCCGC	Broccoli	GTGGCCGCGG	TGTTGAA	CR31905*

ATCCATAGGTCGCCGGTTCGATTCCGGCTCGGAGGA

B

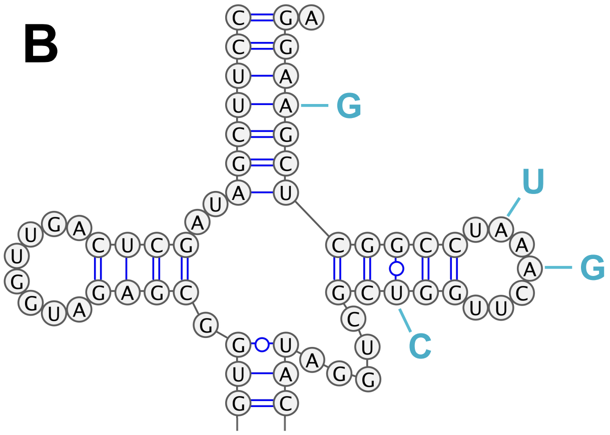

Supplementary Figure S1: Detailed description of reporters used in this study. (A) Table of the specific sequences used in each reporter. Restriction enzyme sites are in red. For the dual reporter, the entire 3' exon sequence is shown. The four mutations are shown in blue. The orange line shows where the probe binds. (B) Partial structure of the CR31905 tRNA molecule, indicating where the mutations were made.

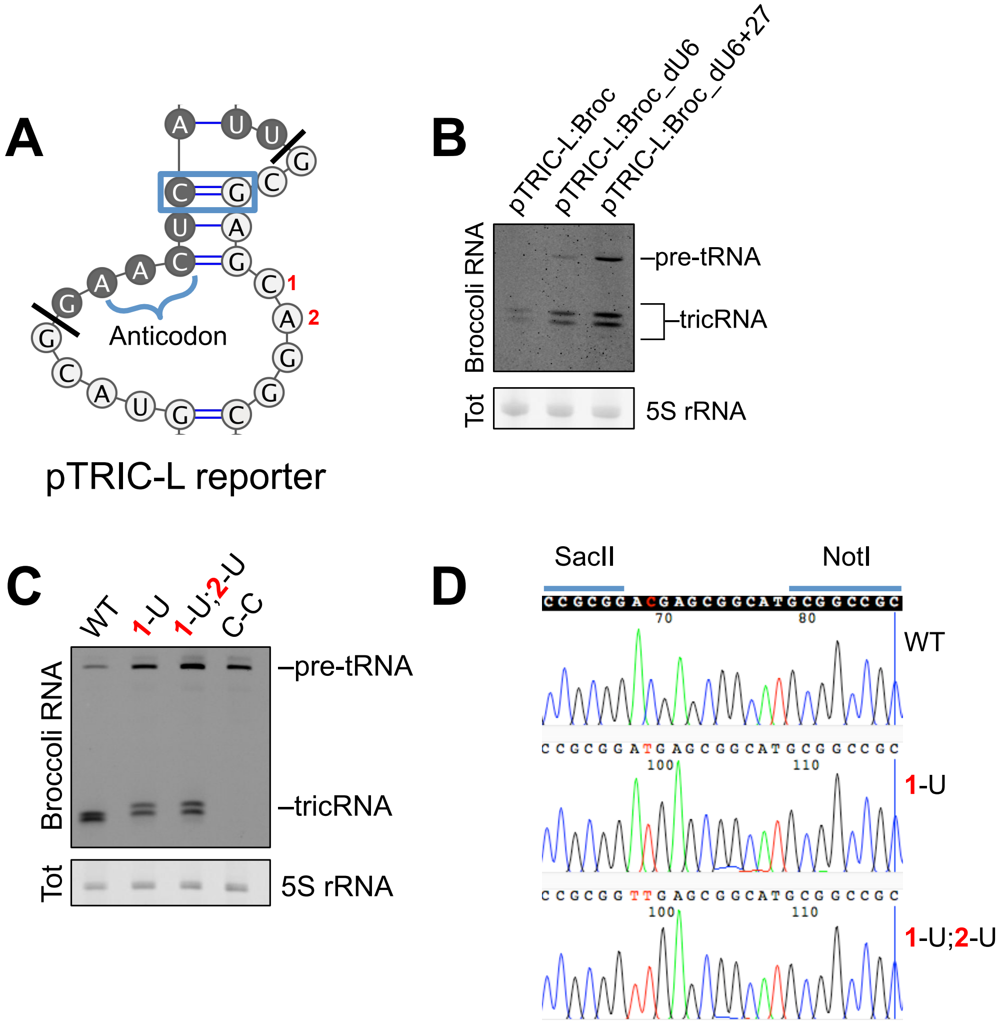

Supplementary Figure S2: Similar trends observed using a different tricRNA reporter. (A) Nucleotide sequence of the BHB-like motif in the pTRIC-L tricRNA reporter (see Fig. S5 for endogenous BHB-like motif of CR31143). The PBP is boxed in blue. Mutated residues are numbered 1 and 2. The darker shaded bases are part of the tRNA, and the lighter shaded bases are part of the intron. (B) In-gel fluorescence assay of RNA from S2 cells transfected with the pTRIC-L:Broc reporter to test expression of the U6 and U6+27 external RNA polymerase III promoters. The pre-tRNA and tricRNA bands are identified. The 5S rRNA band from the EtBr-stained gel is shown as a loading control. (C) In-gel fluorescence assay of RNA from S2 cells transfected with the wild-type and mutant reporters. The pre-tRNA and tricRNA bands are identified. The 5S rRNA band from the EtBr-stained gel is shown as a loading control. (D) Sequence traces of tricRNA junctions from the experiment in (C). The mutations introduced by site-directed mutagenesis (1-U and 1-U;2-U) can be seen in the traces.

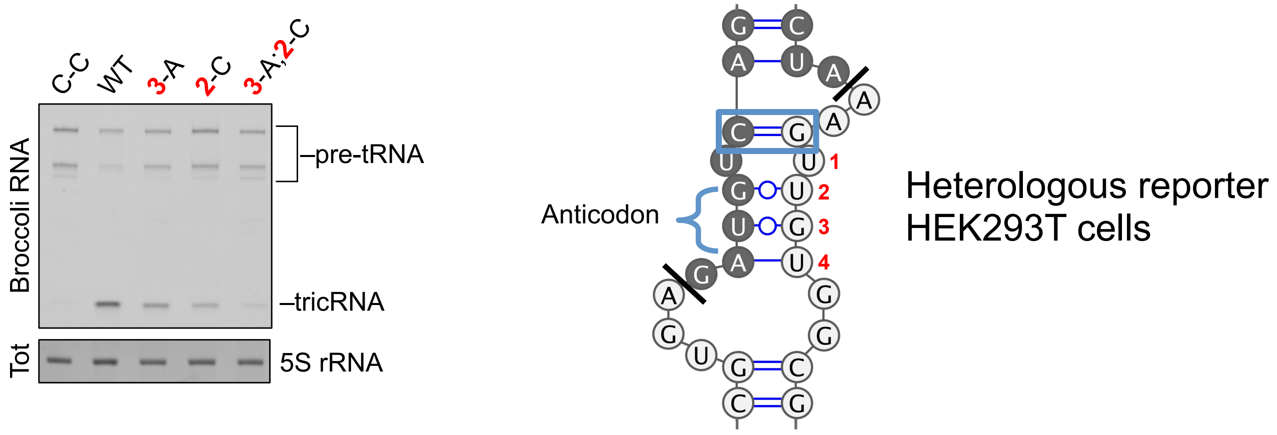

Supplementary Figure S3: mutations in the heterologous tricRNA reporter have similar effects on tricRNA splicing. Left: In-gel fluorescence assay of RNA from HEK293T cells transfected with the heterologous reporter to test expression of various mutant constructs. The pre-tRNA and tricRNA bands are identified. The 5S rRNA band from the EtBr-stained gel is shown as a loading control. Right: Nucleotide sequence of the BHB-like motif in the heterologous reporter. The PBP is outlined in blue. The other base pairs of the helix are numbered 1-4. The darker bases are part of the tRNA, and the lighter bases are part of the intron.

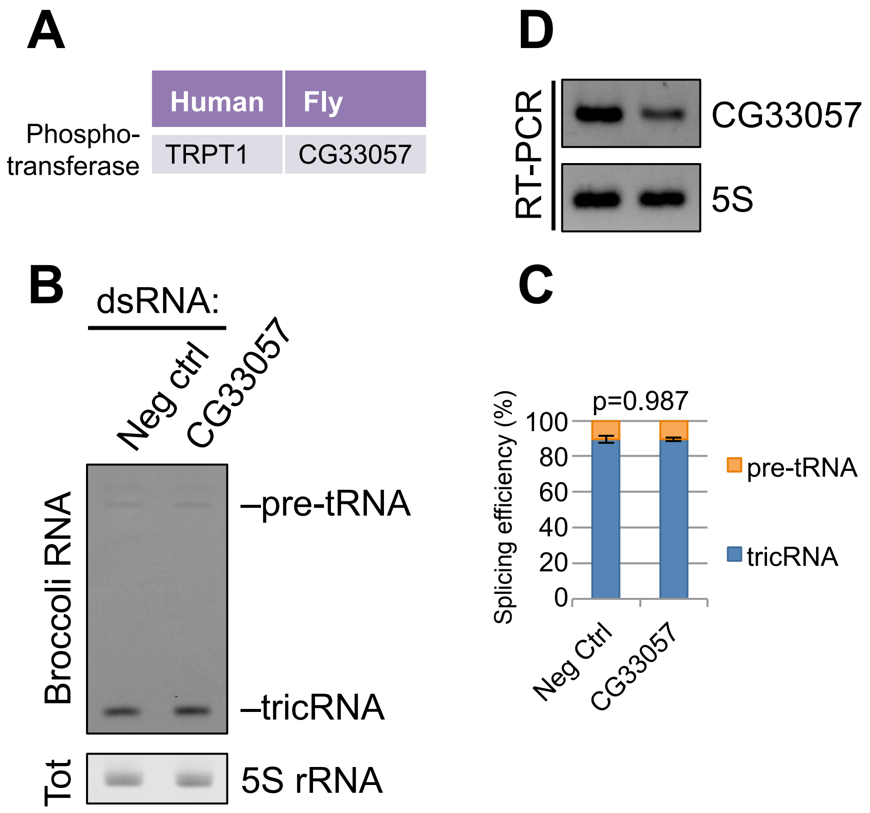

Supplementary figure S4: CG33057 does not participate in reporter tricRNA splicing. (A) *Drosophila* CG33057 is a sequence homolog of human TRPT1. (B) In-gel fluorescence assay of RNA from S2 cells depleted of CG33057. The pre-tRNA and tricRNA bands are identified. The 5S rRNA band from the EtBr-stained gel is shown as a loading control. (C) Quantification of two biological replicates of (B). Error bars denote standard error of the mean. The p-value was calculated using student's t-test. (D) RT-PCR for CG33057 to test knockdown efficiency in S2 cells. RT-PCR for 5S rRNA was used as a control.

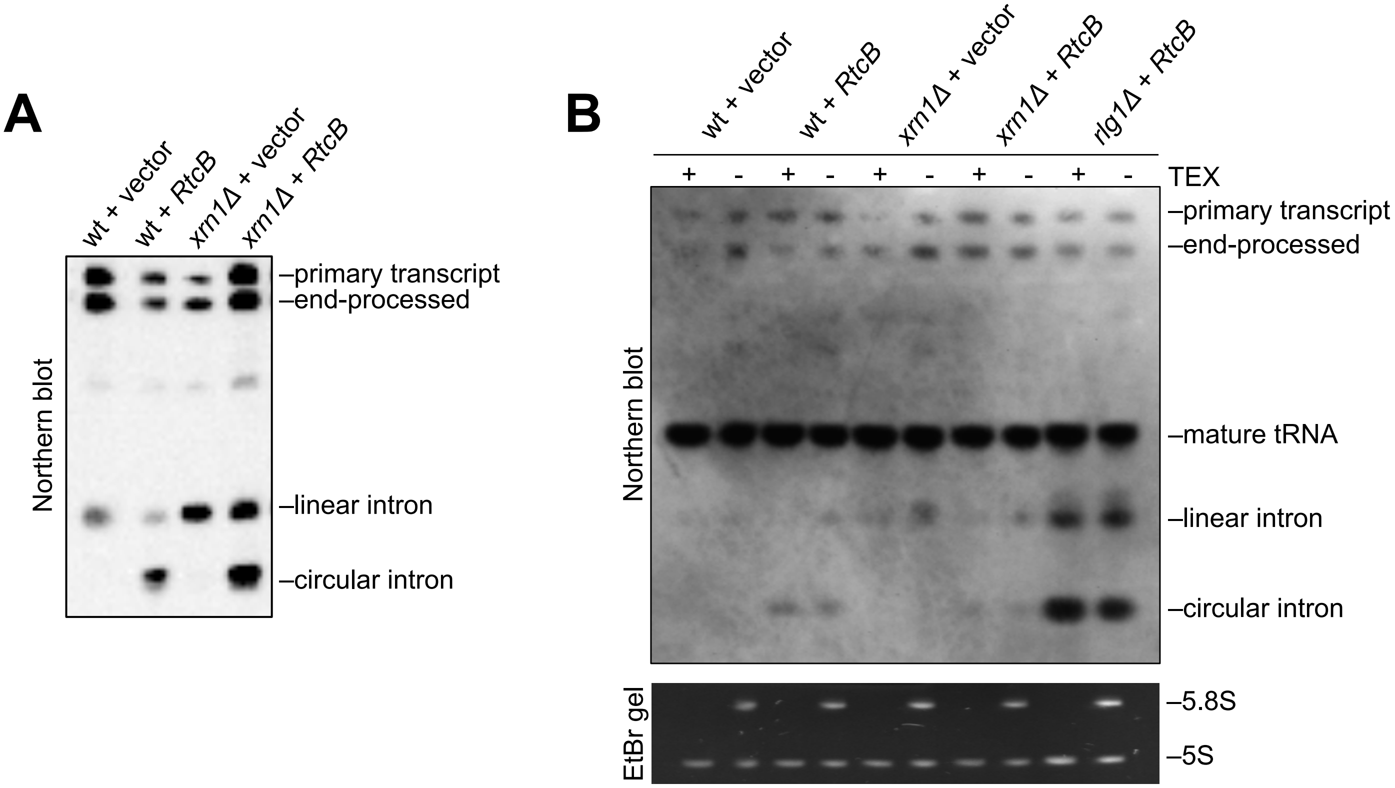

Supplementary Figure S5: Yeast expressing bacterial RtcB generate tricRNAs. (A) Northern blot of RNA from yeast bearing various deletions and enzyme replacements using a probe against tRNA:Ile_{UAU} intron. (B) Northern blot of RNA from yeast bearing various deletions and enzyme replacements using probes for the 5’ exon and intron of the tRNA: Ile_{UAU}. Top: RNA samples were left untreated (-) or treated (+) with terminator exonuclease (TEX). Bottom: EtBr-stained gel showing 5S and 5.8S as a control.

Yeast lacking the healing and sealing enzyme Rlg1/Trl1 are inviable, but this defect can be complemented by RtcB (11). Notably, there is a clear buildup of circular tRNA introns in the the “RtcB replacement” (*rlg1Δ+RtcB*) strain, along with the linear form. TEX (terminator exonuclease) degrades the majority of linear, unstructured RNAs (e.g. 5.8S rRNA is a good substrate whereas 5S rRNA is refractive to cleavage, see bottom panel of S5B). TEX requires a 5' phosphate for its activity, and because Rlg1/Trl1 also contains the kinase activity that normally phosphorylates the 5' end of the excised intron (6), the linear tRNA introns in the RtcB replacement yeast are not phosphorylated on their 5' ends and are thus poor substrates for TEX.

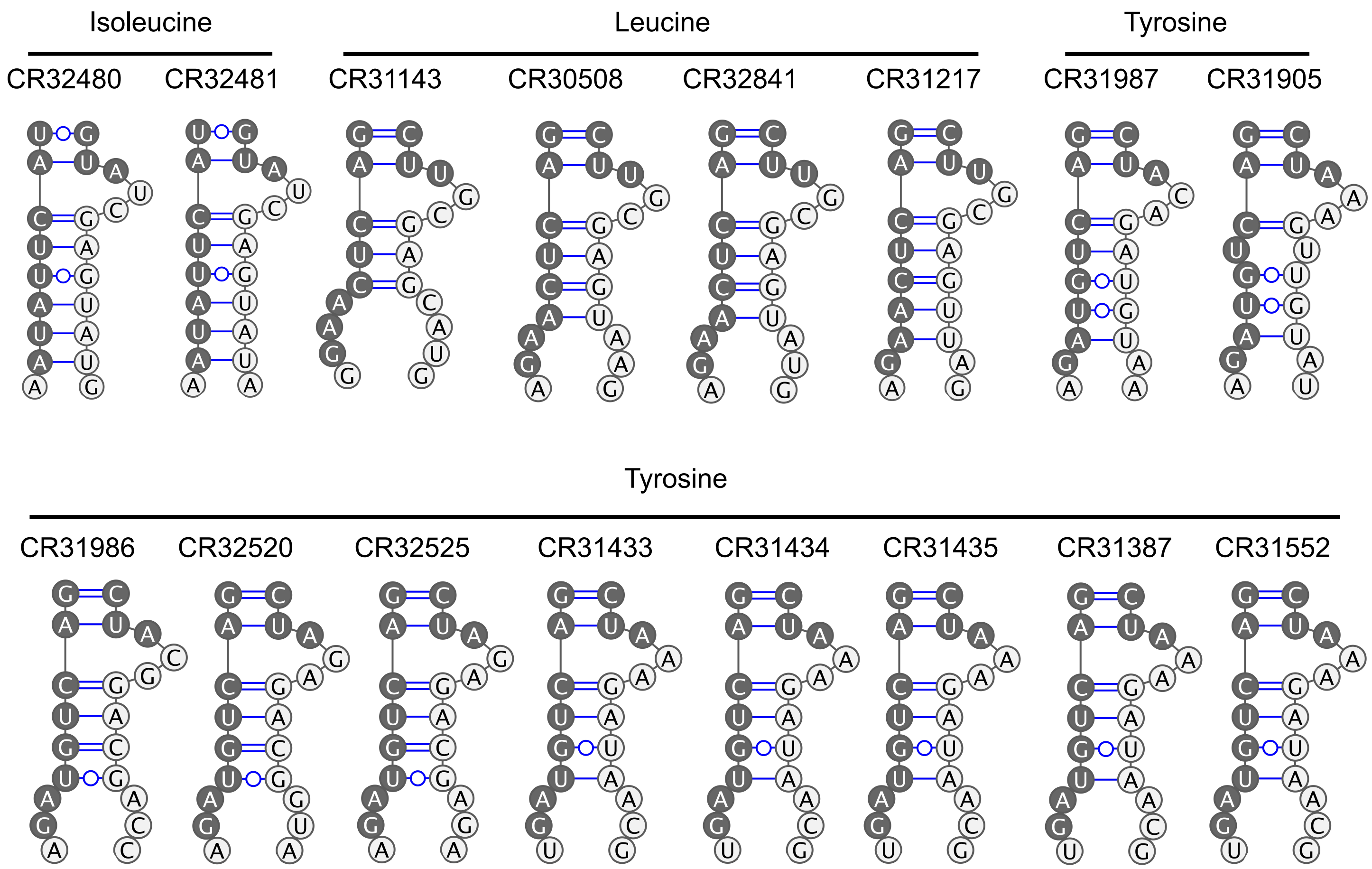

Supplementary Figure S6: Predicted secondary structures of BHB-like motifs for all *Drosophila* intron-containing pre-tRNAs. Sequences of Computed RNA (CR) genes were obtained from FlyBase (http://flybase.org) and the Genomic tRNA Database (http://gtrnadb.ucsc.edu/). CR31905 was discovered after publication of the database. Structures were predicted using Mfold (http://unafold.rna.albany.edu/?q=mfold/RNA-Folding-Form), and structures were drawn using VARNA (http://varna.lri.fr/).

Purpose	Primer name	Sequence 5'-3'
Generating dual reporter	Dual rep_F	CCGGCTCGGAGGATATTATTTTGGGTTTTCTTC
	Dual rep_R	AATCGAACCGGCGACCTATGGATTTCAAC
Northern blot probes	U1	GAATAATCGCAGAGGTCAACTCAGCCGAGGT
	U6	CTTCTCTGTATCGTTCCAATTTTAGTATATGTTCTGCCGAAGCAAGA
	7SK	TAACCCGTCGTCATCCAGTGGAAGGCAG
	Dual reporter probe	TCCGAGCCGGAATCGAACCGG
	S.c. tRNA-Ile 5' exon	TATAAGCACGAAGCTCTAACCACTGAGCTACACGAGC
	S.c. tRNA-Ile intron	CGTTGCTTTTAAAGGCCTGTTTGAAAGGTCTTTGGCACAGAAACTTCGGAAACCGAATGTTGCTAT
Reducing stem length	shortstem5_F	GTATCTGTCGAGTAGAGTGTGGGCTCGCGGTGTTGAAATCCATAGGTCG
	shortstem5_R	GAATATCTGGACCCGACCGTCTCGCACTCTACAGTCCACCGC
	shortstem6_F	GTATCTGTCGAGTAGAGTGTGGGCTCCGCGGTGTTGAAATCCATAGGT
	shortstem6_R	GAATATCTGGACCCGACCGTCTCCGCACTCTACAGTCCACCG
	shortstem7_F	GTATCTGTCGAGTAGAGTGTGGGCTCCCGCGGTGTTGAAATCCATAGGT
	shortstem7_R	GAATATCTGGACCCGACCGTCTCCCGCACTCTACAGTCCACCG
	shortstem8_F	GTATCTGTCGAGTAGAGTGTGGGCTCGCCGCGGTGTTGAAATCCATAGGT
	shortstem8_R	GAATATCTGGACCCGACCGTCTCGCCGCACTCTACAGTCCACCG
	shortstem9_F	GTATCTGTCGAGTAGAGTGTGGGCTCGGCCGCGGTGTTGAAATCCATAGGT
	shortstem9_R	GAATATCTGGACCCGACCGTCTCGGCCGCACTCTACAGTCCACCG
	shortstem10_F	GTATCTGTCGAGTAGAGTGTGGGCTCCGGCCGCGGTGTTGAAATCCATAGGT
	shortstem10_R	GAATATCTGGACCCGACCGTCTCCGGCCGCACTCTACAGTCCACCG
pre-tRNA IVT primers	pre-tRNA_IVT_F	GTATAATACGACTCACTATAGCCTTCGATAGCTCAGTTGGT
	pre-tRNA_IVT_R	TCCTTCGAGCCGGATTTGAA
Proximal base pair mutations	G-C_F	TATCTGTCGAGTAGAGTGTGGGCTCGTGGCCGCGGTGTTCAAATCCATAGGTCGCTGG
	G-C_R	CGAATATCTGGACCCGACCGTCTCGCGGCCGCACTCTACACTCCACCGCTCTACCAACT
	A-U_F	TATCTGTCGAGTAGAGTGTGGGCTCGTGGCCGCGGTGTTTAAATCCATAGGTCGCTGG
	A-U_R	CGAATATCTGGACCCGACCGTCTCGCGGCCGCACTCTACATTCCACCGCTCTACCAACT
	U-A_F	TATCTGTCGAGTAGAGTGTGGGCTCGTGGCCGCGGTGTTAAAATCCATAGGTCGCTGG
	U-A_R	CGAATATCTGGACCCGACCGTCTCGCGGCCGCACTCTACAATCCACCGCTCTACCAACT
	G-U_F	TATCTGTCGAGTAGAGTGTGGGCTCGTGGCCGCGGTGTTTAAATCCATAGGTCGCTGG
	G-U_R	CGAATATCTGGACCCGACCGTCTCGCGGCCGCACTCTACACTCCACCGCTCTACCAACT
	U-G_F	TATCTGTCGAGTAGAGTGTGGGCTCGTGGCCGCGGTGTTGAAATCCATAGGTCGCTGG
	U-G_R	CGAATATCTGGACCCGACCGTCTCGCGGCCGCACTCTACAATCCACCGCTCTACCAACT
	C-C_F	CCGCGGTGTTCAAATCCATAGG
	C-C_R	CCACGAGCCCACACTCTA
	Dual rep C-C_F	CCGGCTCGGAGGATATTATTTTGGGTTTTCTTC
	Dual rep C-C_R	AATCGAACCGGCGACCTATGGATTTGAAC
Pairing the helix	3-UA_F	TGGCCGCGGTATTGAAATCCA
	3-UA_R	CGAGCCCACACTCTACTC
	2-GC_F	GGCCGCGGTGCTGAAATCCAT
	2-GC_R	ACGAGCCCACACTCTACTC
	1-UA_F	GCCGCGGTGTAGAAATCCATA
	1-UA_R	CACGAGCCCACACTCTAC
	3-UA;2-GC_F	TGGCCGCGGTACTGAAATCCATAG
	3-UA;2-GC_R	CGAGCCCACACTCTACTC
	2-GC;1-UA_F	GGCCGCGGTGCAGAAATCCATAG
	2-GC;1-UA_R	ACGAGCCCACACTCTACT
	3-UA;2-GC;1-UA_F	TGGCCGCGGTACAGAAATCCATAG
	3-UA;2-GC;1-UA_R	CGAGCCCACACTCTACTC
Unpairing the helix	4-AA_F	GTGGCCGCGGAGTTGAAATCC
	4-AA_R	GAGCCCACACTCTACTCG
	4-AA;3-UU_F	GTGGCCGCGGATTTGAAATCCATAG
	4-AA;3-UU_R	GAGCCCACACTCTACTCG
	3-UU;2-GG_F	TGGCCGCGGTTGTGAAATCCATAG
	3-UU;2-GG_R	CGAGCCCACACTCTACTC
	4-AA;3-UU;2-GG_F	GTGGCCGCGGATGTGAAATCCATAGGTC
	4-AA;3-UU;2-GG_R	GAGCCCACACTCTACTCG
	4-AA;3-UU;2-GG;1-UA_F	GTGGCCGCGGATGAGAAATCCATAGG
	4-AA;3-UU;2-GG;1-UA_R	GAGCCCACACTCTACTCG
Human tricRNA reporter *cis* element mutations	C-C_F	TGTTCAAATCCATAGGTCGCTGGTTCAAATCCGGC
	C-C_R	CCGCGGCCACGAGCC
	4-AA_F	AGTTGAAATCCATAGGTCGCTGGTTCAAATCCGGC
	4-AA_R	CCGCGGCCACGAGCC
	3-UA_F	TATTGAAATCCATAGGTCGCTGGTTCAAATCCGGC
	3-UA_R	CCGCGGCCACGAGCC
	2-GC_F	TGCTGAAATCCATAGGTCGCTGGTTCAAATCCGGC
	2-GC_R	CCGCGGCCACGAGCC
	3-UA;2-GC_F	TACTGAAATCCATAGGTCGCTGGTTCAAATCCGGC
	3-UA;2-GC_R	CCGCGGCCACGAGCC

Supplementary Table 1: List of oligonucleotides used in this study

Purpose	Primer name	Sequence 5'-3'
Making PCR products for *in vitro* transcription of *Drosophila* processing factors	TSEN2_F	TAATACGACTCACTATAGGGGGTATTAAGTTCGGCGGTGATTTTCG
	TSEN2_R	TAATACGACTCACTATAGGGCTTCTTAGGCGGTTGGACAGTC
	TSEN15_F	TAATACGACTCACTATAGGGGCTGTTGTGAATCTGGCACAGA
	TSEN15_R	TAATACGACTCACTATAGGGATCATCGGGGCTTACAGTTGTG
	TSEN34_F	TAATACGACTCACTATAGGGCGAACACAAAAGGCTGGAGTC
	TSEN34_R	TAATACGACTCACTATAGGGACTCCTCAAGTTTCTCGGCA
	TSEN54_F	TAATACGACTCACTATAGGCGTTTACAACTGGAGTACTGTGGTTT
	TSEN54_R	TAATACGACTCACTATAGGGCACCAAATCGAACTTTTCAAAGATC
	Ddx1_F	TAATACGACTCACTATAGGGCGCCTAACGCCCCTCA
	Ddx1_R	TAATACGACTCACTATAGGGCGGGTCGACTAGACAGACGA
	Archease_F	TAATACGACTCACTATAGGGCCACGGATGGGGATCGT
	Archease_R	TAATACGACTCACTATAGGGAATGTCAATTATCACGAACACCTCGTAGT
	RtcB_F	TAATACGACTCACTATAGGACGCCGAGATCCAGGTGG
	RtcB_R	TAATACGACTCACTATAGGAACGCTTGACGGGTGAGGAATG
	CG33057_F	TAATACGACTCACTATAGGGCGTTCCCGATTTGCAGAAGCA
	CG33057_R	TAATACGACTCACTATAGGGTCCGCCAGCACCTTTTCC
	Zucchini_F	TAATACGACTCACTATAGGGAGCAAGCGAGAGAAGGCAAG
	Zucchini_R	TAATACGACTCACTATAGGGCCAAGAGCCGTCCAGTTTACG
	Smg6_F	TAATACGACTCACTATAGGGATTGCTGGGCTGAGCTAACAA
	Smg6_R	TAATACGACTCACTATAGGGTGTCCACGAACTTGAGAATGTCC
	Dis3_F	TAATACGACTCACTATAGGGCTTGCCGAAAATGCCCTGGACAATTA
	Dis3_R	TAATACGACTCACTATAGGGATCGCTCAACACCGCCAACC
	Clipper_F	TAATACGACTCACTATAGGGGGACCGGACGATCGTGT
	Clipper_R	TAATACGACTCACTATAGGGCCGGAGTGTCCAGAGTAGC
	Neg ctrl_F	TAATACGACTCACTATAGGGTTCAACATCGTGGCCGTGGC
	Neg ctrl_R	TAATACGACTCACTATAGGGGTTGGCAAGCCCTTTGAGGCA
Drosophila processing factor RT-PCR primers	TSEN2_F	TGGTTGTTTTGGCAAGGGAAGCA
	TSEN2_R	ACCTCCACAAACATGCAGAAGTCC
	TSEN15_F	GATTTGACCGCCGCTTTGGG
	TSEN15_R	GCCTTTGTGTGCAGCACAGG
	TSEN34_F	GGTACTGGCTTCGTTTTCAACGTGG
	TSEN34_R	GTTTCAAATTTACTGAGCTCCACGGGC
	TSEN54_F	GGAGCTTAAACGAGCGCAGGAGTA
	TSEN54_R	GCTTTCCCTGCTCACTGTATCCGAA
	Ddx1_F	CAGACGAATTGGACTGGACCCTG
	Ddx1_R	GGTCTCCCACACGATTTGCAGAA
	Arch_F	CTGGATCACACGGCGGATGTTC
	Arch_R	CTCTCCAGATCATCTCCATGCGC
	RtcB_F	CTTCCCGCCACACCATCC
	RtcB_R	CACGACCCGCTCCGTG
	CG33057_F	GGAATCACGATCCGTGCTGATGG
	CG33057_R	CGAAGAGTATATCGTTGCTTGGCATCC
	Zucchini_F	CCTCAGAGGTGATTTGGAAGCTGG
	Zucchini_R	CGACACTTGTGGTTTCTGGGATCC
	Smg6_F	CGAGCACAACAAACCCAAAACGTCA
	Smg6_R	GCAAAGTCTTCTTCTGGGAGACACTG
	Dis3_F	CGAGCACCACAAGGAAACCTATGC
	Dis3_R	CTCTTTAGAGGATTGCGAGGCCTG
	Clipper_F	CATCACCAGGAACGGACAGGAG
	Clipper_R	GTAGCACTCGGGCATCTTGGT
	5S_F	AACAACACGCGGTGTTCCCAAGC
	5S_R	GCCAACGACCATACCACGCTGAA
Gateway cloning *Drosophila* endonucleases	Dis3_N_F	GGGGACAAGTTTGTACAAAAAAGCAGGCTTCCAAACTTTACGCGAATTTAC
	Dis3_N_R	GGGGACCACTTTGTACAAGAAAGCTGGGTNTTACTTCTTTTTCTTATCCTTCT
	Dis3_C_F	GGGGACAAGTTGGTACAAAAAAGCAGGCTATGCAAACTTTACGCGAA
	Dis3_C_R	GGGGACCACTTTGTACAAGAAAGCTGGGTCCTTCTTTTTCTTATCCTTCTTTGTC
	Clp_N_F	GGGGACAAGTTTGTACAAAAAAGCAGGCTTCGACATCCTTTTGGCCAAC
	Clp_N_R	GGGGACCACTTTGTACAAGAAAGCTGGGTNCTACTTATGACTATGTTGGTTAGAG
	Clp_C_F	GGGGACAAGTTGGTACAAAAAAGCAGGCTATGGACATCCTTTTGGCC
	Clp_C_R	GGGGACCACTTTGTACAAGAAAGCTGGGTCCTTATGACTATGTTGGTTAGAGAGG
Yeast RT-PCR primers	S.c. Ile_F	TGCTTTTAAAGGCCTGTTTGAAAGG
	S.c. Ile_R	GCAACATTCGGTTTCCGAAGTTTCT

Supplementary Table 1 continued: List of oligonucleotides used in this study
